# Supplementary material for: Social signaling via bioluminescent blinks determines nearest neighbor distance in schools of flashlight fish Anomalops katoptron
Source: Sci Rep. 2021 Mar 19;11:6431. doi: 10.1038/s41598-021-85770-w (PMC7979757; doi:10.1038/s41598-021-85770-w)
Supplement: Supplementary file 1 — Supplementary Information 1. [file 41598_2021_85770_MOESM1_ESM.pdf]

Supplementary Figures for the Manuscript:

Social signaling via bioluminescent blinks determines nearest neighbor distance in schools of flashlight fish *Anomalops katoptron*

Peter Jägers[1], Louisa Wagner[1], Robin Schütz[1], Maximilian Mucke[1], Budiono Senen[2]

Gino V. Limmon[3,4], Stefan Herlitze[1#], Jens Hellinger[1]

[1]Department of General Zoology and Neurobiology, Institute of Biology and Biotechnology, Ruhr-University Bochum, 44780 Bochum, Germany

[2]Fisheries College Hatta-Syahrir, Banda Naira, Malukuh Tengah, Indonesia

[3]Department of Aquatic Resources Management, Faculty of Fisheries and Marine Science, Pattimura University Ambon, Indonesia

[4]Maritime and Marine Science Center for Excellence of Pattimura University Ambon, Indonesia

#corresponding author: sxh106@gmail.com

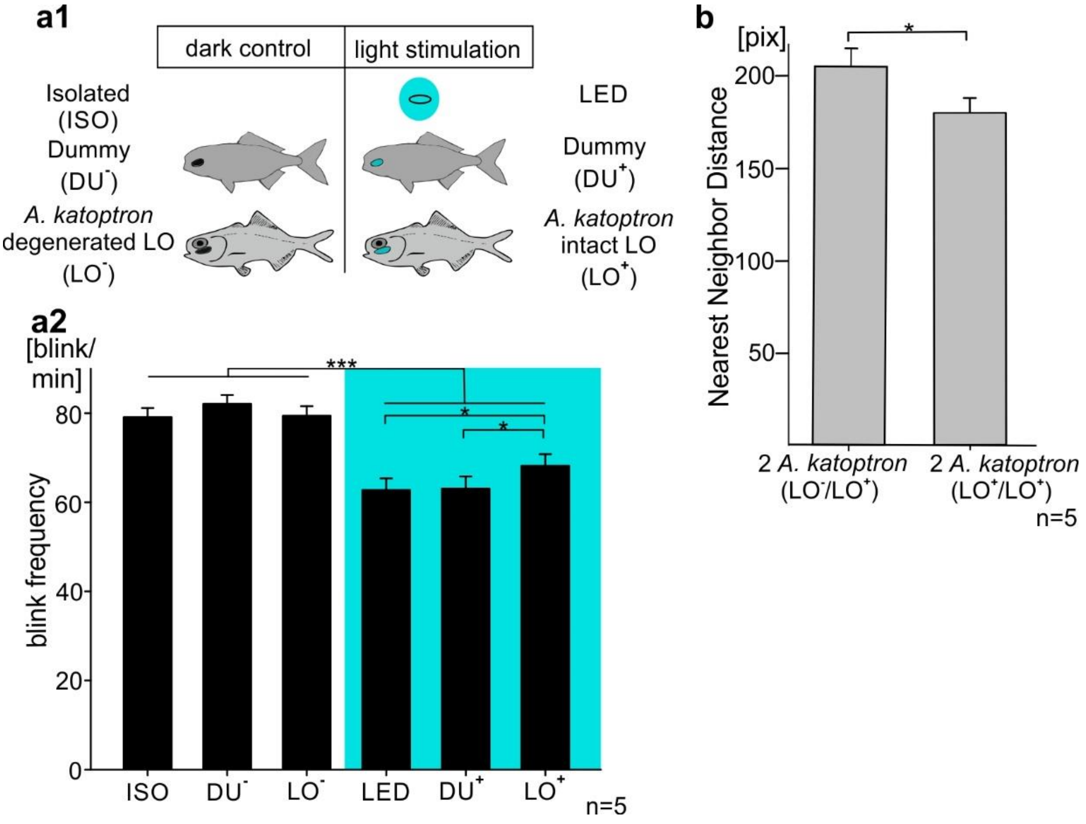

Figure S1. Blink behavior of *A. katoptron* during exposure to different artificial light stimuli and their orientation towards conspecifics.

(a1) Natural and artificial light stimulation used in the experiments to investigate the blink behavior of *A. katoptron*. Two different types (light stimulation or dark controls) were presented to isolated individuals, i.e. an isolated LED and a fish dummy, equipped with a LED at the position of the light organ. Artificial lights had the same size, intensities and emitted 1 Hz light pulses (equally distributed LED on- and off- times). Experiments were repeated 5 times with five individuals independently and values are given as mean (± SEM). The behavioral responses to the artificial lights were compared to responses to *A. katoptron* with an intact light organ (LO^+) and a degenerated light organ (LO^-).

(a2) Blink frequencies of isolated *A. katoptron* during exposure to dark controls (left, white background) and light stimulation (right, blue background). Blink frequencies were reduced in the presence of light-stimuli. LED and the fish dummy light stimuli reduced the blink frequency more than the conspecifics.

(b) *A. katoptron* show a closer mean (± SEM) orientation towards its neighbors when both specimen display intact light organs (LO^+/LO^+).

Statistical significance was evaluated with RM ANOVA.

Significance values are reported as: *$p < 0.05$, **$p < 0.01$, ***$p < 0.001$. Error bars indicate SEM.

Figures were created with SigmaPlot 12.0 (www.sigmaplot.co.uk) and processed with CorelDraw Graphics Suite 2017 (www.coreldraw.com).

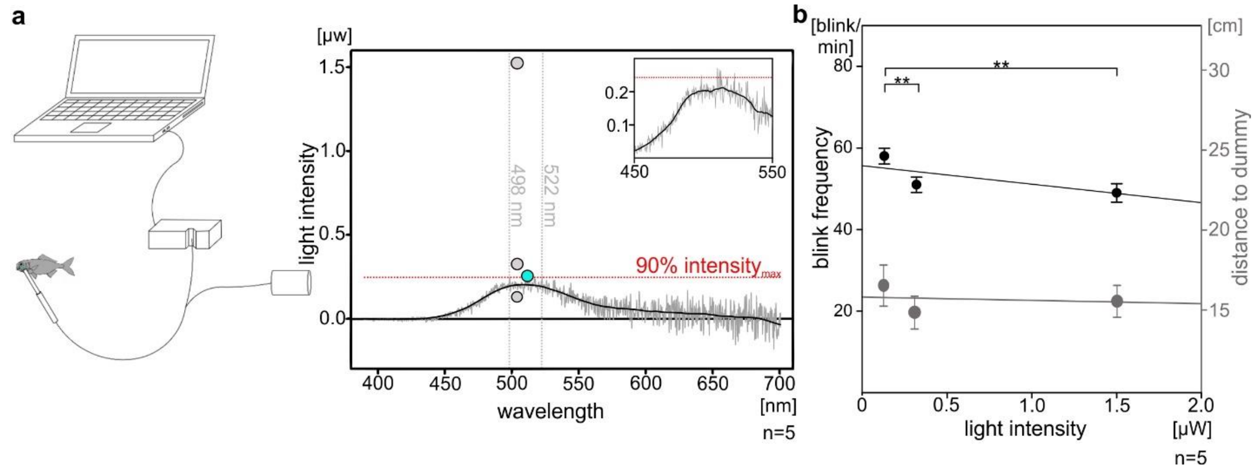

Figure S2. Spectrometric measurements of the light intensities emitted by light organs of *A. katoptron* in comparison to LEDs and the dependency of blinking behavior on different light intensities.

(a) Spectrometric measurement of the light organ (n=5) intensity of *A. katoptron* in comparison to LED light. Diagram of the experimental setup for the spectrometric measurements (left). Intensities were measured with a spectrometer (Flame S-UV-VIS-ES, Ocean Optics, USA). The spectroscopic probe was placed in front of the light organs of fixed individuals. Each light organ was measured five times and mean intensity averaged. Example trace of the spectrometric measurement of the light intensities emitted by the light organs measured in the range between 400 – 700 nm wavelength (right). Solid black line represents the smoothed intensities. Smoothed intensities were calculated with a polynomial regression and weights computed from the Gaussian density function in SigmaPlot (SigmaPlot 12.0). Red dotted lines indicate 90 % intensity level. Inset shows light intensities between 450 – 550 nm. The green dot indicates the maximum intensity observed in *A. katoptron* (λ max = 510 nm; 0,27 µW). Grey dots indicate three different LED intensities of 0.133, 0.328 & 1.523 µW at 504 nm wavelength, which were presented to isolated flashlight fish (*A. katoptron*) to investigate impact on blink frequency.

(b) Blink frequency responses of *A. katoptron* and distance to dummy triggered by the three distinct LED intensities detected at 504 nm wavelengths as shown in A. Experiments were repeated 5 times independently and values are given as mean (± SEM). Blink frequencies of *A. katoptron* were decreased with increasing intensities of the LED.

Statistical significance was evaluated with RM ANOVA.

Significance values reported as: **p < 0.01. Error bars indicate ± SEM.

Figures were created with SigmaPlot 12.0 (www.sigmaplot.co.uk) and processed with CorelDraw Graphics Suite 2017 (www.coreldraw.com).

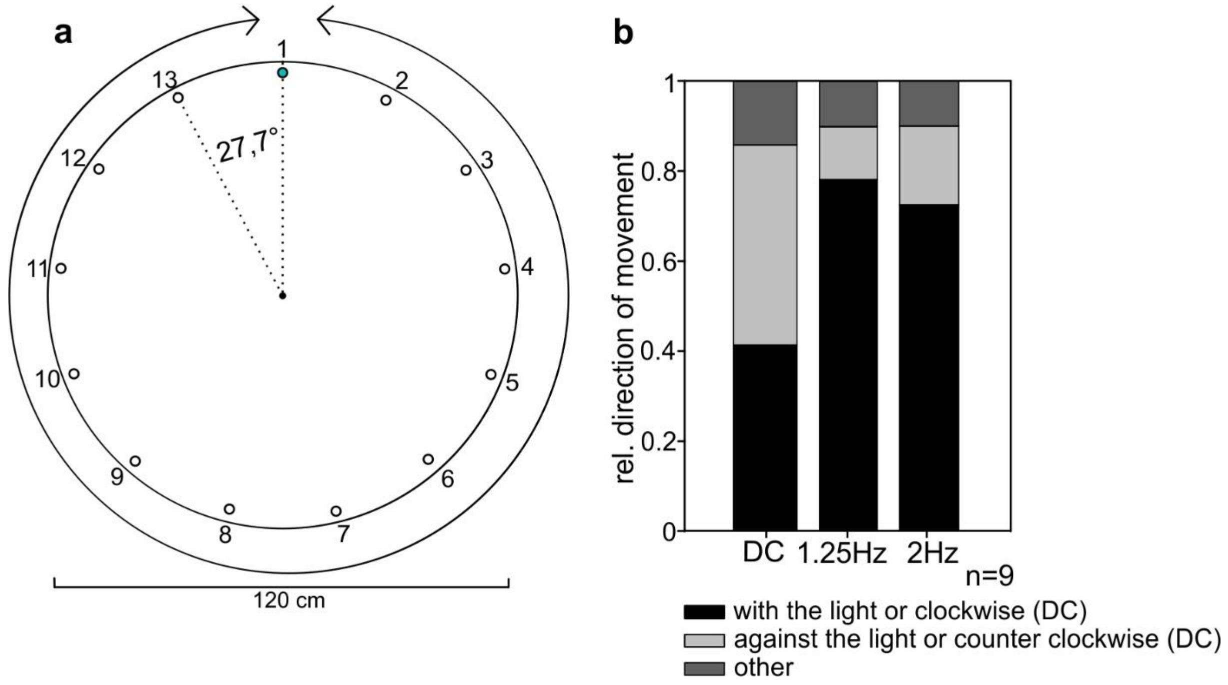

Figure S3. ***A. katoptron*** **follow moving light stimuli.**

(a) Experimental setup with 13 wall mounted LEDs that were triggered consecutively counter- or clockwise. Intervals between 300 ms light emittance were 200 or 500 ms (travelling speed of light: 200 ms, 0.58 m/s; 500 ms, 0.36 m/s). Additionally, we performed a control without light stimulation (DC, dark control). Each fish was tested for 60 seconds.

(b) Relative direction of *A. katoptron* following artificial light sources. Flashlight fish show a high motivation to follow the direction of light (1.25 & 2 Hz). In the control experiment (DC) the fish swims equally into both (counter clockwise or clockwise) directions.

Figures were created with SigmaPlot 12.0 (www.sigmaplot.co.uk) and processed with CorelDraw Graphics Suite 2017 (www.coreldraw.com).

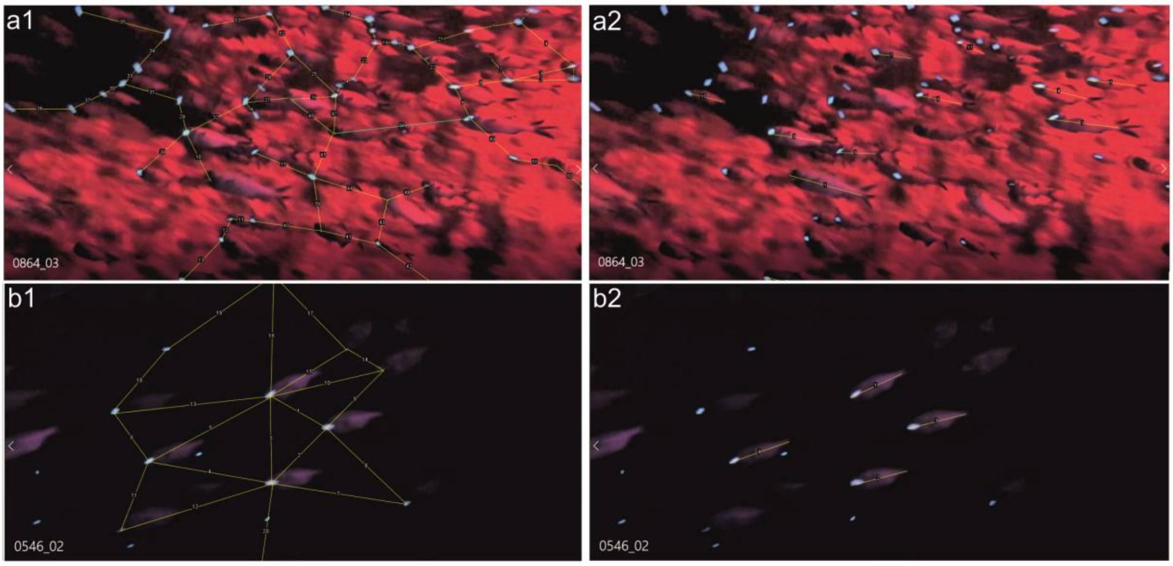

Figure S4. Analyzing the nearest neighbor distance in schools of *A. katoptron* (Ambon, Maluku, Indonesia).

(a) Flashlight fish *A. katoptron* were illuminated with diving torches (300 lumen red light; Codylight 1500; Germany) to trigger avoidance reactions. For every screenshot, we estimated the fish standard length (SL) as reference (a2) We connected light organs (a1) of individuals that seemed to be neighbors to determine their distances. 46 screenshots were analyzed.

(b) Groups of *A. katoptron* while schooling on the reef flat were illuminated with IR-torches and recorded with an infrared camera. Networks connect light organs of potential neighbors (b1) and standard length (SL) was estimated as reference (b2). 37 screenshots were analyzed.

Screenshots were processed with CorelDraw Graphics Suite 2017 (www.coreldraw.com).
